# IgA Nephropathy Patient Baseline Characteristics in the Sparsentan PROTECT Study

**DOI:** 10.1016/j.ekir.2023.02.1086

**Published:** 2023-03-04

**Authors:** Jonathan Barratt, Brad Rovin, Muh Geot Wong, Charles E. Alpers, Stewart Bieler, Ping He, Jula Inrig, Radko Komers, Hiddo J.L. Heerspink, Alex Mercer, Irene L. Noronha, Jai Radhakrishnan, Michelle N. Rheault, William Rote, Howard Trachtman, Hernán Trimarchi, Vlado Perkovic, PROTECT investigators

**Affiliations:** 1 Department of Cardiovascular Sciences, University of Leicester General Hospital, Leicester, UK; 2 Division of Nephrology, Ohio State University Wexner Medical Center, Columbus, Ohio, USA; 3 Department of Renal Medicine, Concord Repatriation General Hospital, Concord, New South Wales, Australia; 4 Concord Clinical School, University of Sydney, Concord, New South Wales, Australia; 5 Department of Laboratory Medicine and Pathology, University of Washington, Seattle, Washington, USA; 6 Travere Therapeutics Inc., San Diego, California, USA; 7 Department of Clinical Pharmacy and Pharmacology, University Medical Center Groningen, University of Groningen, Groningen, the Netherlands; 8 The George Institute for Global Health, University of New South Wales, Sydney, New South Wales, Australia; 9 JAMCO Pharma Consulting, Stockholm, Sweden; 10 Laboratory of Cellular, Genetic, and Molecular Nephrology, Division of Nephrology, University of São Paulo School of Medicine, São Paulo, Brazil; 11 Division of Nephrology, Columbia University, New York, New York, USA; 12 Division of Pediatric Nephrology, University of Minnesota Medical School, Minneapolis, Minnesota, USA; 13 Division of Nephrology, Department of Pediatrics, University of Michigan, Ann Arbor, Michigan, USA; 14 Nephrology Service, Hospital Británico de Buenos Aires, Buenos Aires, Argentina; 15 Faculty of Medicine and Health, University of New South Wales Sydney, Sydney, New South Wales, Australia

**Keywords:** dual endothelin angiotensin receptor antagonist, ethnicity, immunoglobulin A nephropathy, race, randomized controlled clinical trial, sparsentan

## Abstract

**Introduction:**

Sparsentan is a novel single-molecule dual endothelin angiotensin receptor antagonist with hemodynamic and anti-inflammatory properties and is not an immunosuppressant. The ongoing phase 3 PROTECT trial examines sparsentan in adults with IgA nephropathy (IgAN).

**Methods:**

The PROTECT trial (NCT03762850) is a multicenter, international, randomized, double-blind, parallel-group, active-controlled study. The efficacy and safety of sparsentan versus the active control irbesartan is being evaluated in adults with biopsy-proven IgAN and proteinuria ≥1.0 g/d despite maximized treatment with an angiotensin-converting enzyme inhibitor (ACEi) and/or angiotensin receptor blocker (ARB) for at least 12 weeks. Blinded and aggregated baseline characteristics are reported descriptively and compared to contemporary phase 3 trials with patients with IgAN.

**Results:**

The primary analysis population includes 404 patients who were randomized and received study drug (median age, 46 years). Enrolled patients were from Europe (53%), Asia Pacific (27%), and North America (20%). Baseline median urinary protein excretion was 1.8 g/d. The range of estimated glomerular filtration rate (eGFR) was broad with the largest proportion of patients (35%) in chronic kidney disease (CKD) stage 3B. Before transitioning to study medication, mean systolic/diastolic blood pressure was 129/82 mm Hg, with the majority of patients (63.4%) receiving the maximum labeled ACEi or ARB dose. Patients in Asian versus non-Asian regions included a higher percentage of females, had lower blood pressures, and included lower proportions of patients with a history of hypertension and baseline antihypertensive treatment.

**Conclusions:**

Patient enrollment in PROTECT, with differing racial backgrounds and across CKD stages, will allow for important characterization of the treatment effect of sparsentan in patients with IgAN with proteinuria at high risk of kidney failure.

## Graphical abstract

**Figure:**
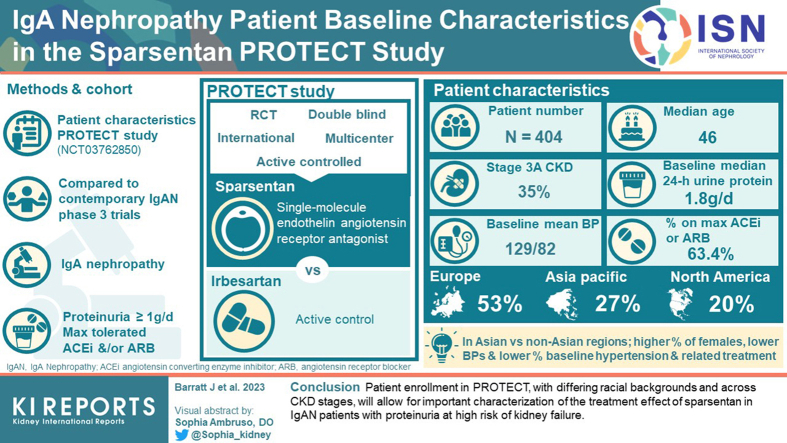

IgAN is the most prevalent primary glomerulopathy worldwide, with an estimated incidence of approximately 2.5 per 100,000 individuals per year.^1^^,^^2^ IgAN is a heterogenous disease with variable disease progression, including differences in progression across geographic regions and race.^3^ Among patients diagnosed with IgAN, 20% to 40% progress to kidney failure requiring dialysis or kidney transplantation within 10 to 20 years,^4^, ^5^, ^6^ with serious effects on quality of life and mortality.^7^, ^8^, ^9^ The standard of care for patients with IgAN includes renin-angiotensin-aldosterone system inhibition (RAASi) with ACEis or ARBs, control of blood pressure, and lifestyle modifications.^10^ It is recommended that patients identified as being at high risk of progressing to kidney failure based on persistent proteinuria >0.75 to 1 g/d despite ≥90 days of maximized standard of care should be considered for additional treatment, including participation in a clinical trial.^10^

In parallel with a variety of beneficial pleiotropic nephroprotective effects of RAASi, used as part of standard of care, selective endothelin type A receptor antagonists have been shown to have hemodynamic, anti-inflammatory, antifibrotic, and podocyte-protective effects in models of kidney diseases.^11^ Therefore, there is a strong rationale for a therapeutic approach with simultaneous antagonism of these 2 pathways that act in tandem. Importantly, the actions of RAASi and endothelin type A receptor antagonists in combination have demonstrated additive benefits in experimental models of kidney disease^12^^,^^13^ and in patients with both diabetic^14^^,^^15^ and nondiabetic CKD, including patients with IgAN.^16^

Sparsentan is a novel nonimmunosuppressive single molecule that is a dual-acting, highly selective antagonist of both endothelin type A receptor and the angiotensin II subtype 1 receptor.^13^^,^^17^ In DUET, a phase 2 randomized controlled clinical trial, treatment with sparsentan significantly reduced proteinuria compared to the active control ARB irbesartan in patients with focal segmental glomerulosclerosis.^18^ Moreover, the ongoing open-label extension of DUET has demonstrated sustained proteinuria reduction and a favorable safety profile in patients who continue to receive sparsentan.^19^^,^^20^ In parallel with the development in focal segmental glomerulosclerosis, sparsentan is being studied as a therapeutic for IgAN. The ongoing PROTECT study is testing the long-term antiproteinuric and nephroprotective efficacy and safety of sparsentan compared with the active control irbesartan in 404 adults with IgAN.^21^ Here we report the blinded and aggregated baseline characteristics of patients enrolled in the phase 3 PROTECT trial, including comparisons of patients recruited in Asian and non-Asian geographic regions. Moreover, the baseline characteristics of patients in PROTECT are compared to those of participants included in other recent phase 3 trials involving participants with IgAN.

## Methods

### Study Design

The PROTECT trial (EudraCT number: 2017-004605-41; US ClinicalTrials.gov identifier: NCT03762850) is a phase 3, multicenter, international, randomized, double-blind, parallel-group, active control study designed to evaluate the efficacy and safety of sparsentan (400 mg/d) versus the active control irbesartan (300 mg/d) in adults with IgAN who continue to have persistent, overt proteinuria despite receiving maximized treatment with an ACEi and/or ARB. Details of the study methods have been previously reported^21^ and are briefly reviewed below.

The study duration of 270 weeks includes a double-blind period of 114 weeks (final patient visit expected in the second half of 2023) followed by an open-label extension period of up to 156 weeks. Eligible patients met the following key inclusion criteria at screening: male or female aged ≥18 years; biopsy-proven IgAN (the biopsy may have been performed at any time in the past); 24-hour urine protein excretion value ≥1.0 g/d; eGFR ≥30 ml/min per 1.73 m^2^; on a stable dose of ACEi and/or ARB therapy for at least 12 weeks before screening that is both the patient’s maximum tolerated dose and at least one-half of the maximum labeled dose (MLD); and systolic blood pressure ≤150 mm Hg, and diastolic blood pressure ≤100 mm Hg. Key exclusion criteria were as follows: IgAN secondary to another condition or IgA vasculitis; cellular glomerular crescents present in >25% of glomeruli on kidney biopsy within 6 months before screening; a cause of CKD in addition to IgAN; administration of any systemic immunosuppressive medications (including corticosteroids) for >2 weeks within 3 months before screening; and significant cerebrovascular, cardiovascular (including New York Heart Association Class II–IV heart failure), or hepatic conditions.

### Procedures

Enrolled patients discontinued RAASi and any other prohibited concomitant medications (e.g., sodium-glucose cotransporter-2 inhibitors) before the randomization (day 1) visit and completed comprehensive baseline evaluations and clinical laboratory tests. Routine blood and urine samples for laboratory assessments were analyzed at a central laboratory (Q2 Solutions, Valencia, CA). eGFR was determined using the Chronic Kidney Disease Epidemiology formula.^22^ Safety evaluations included changes from baseline in body weight, vital signs, physical examinations, peripheral edema, clinical laboratory parameters, and the incidence of treatment-emergent adverse events. Patients initiated study treatment at one-half the study drug target dose (i.e., 200 mg/d sparsentan; 150 mg/d irbesartan) for the first 2 weeks and then received the target dose (400 mg/d sparsentan; 300 mg/d irbesartan) following dose tolerance evaluation. Patients without dose tolerance (e.g., asymptomatic blood pressure values ≤100/60 mm Hg or clinical symptoms of orthostatic hypotension) continued receiving the initial dose of study drug. Dose titrations (up or down) were permitted at any time at the investigator’s discretion.

### End Points and Assessments

The primary efficacy end point of the PROTECT trial is the change from baseline (day 1) in urine protein-to-creatinine ratio on the basis of a 24-hour urine sample at week 36. Key secondary efficacy endpoints include chronic eGFR slope over 1 and 2 years (6–58 weeks and 6–110 weeks, respectively) and total eGFR slope over the full double-blind treatment period of 110 weeks, as well as a variety of proteinuria variables up to week 114.

### Data Analysis

In this report of the baseline characteristics of patients enrolled in the PROTECT trial, the blinded and aggregated data of the enrolled patients in the primary analysis population are summarized descriptively. The primary analysis population includes the patients who at the time of data extraction were randomized and had taken at least 1 dose of the assigned treatment. Baseline is defined as the last nonmissing observation on or before the start of dosing. Mean and SD are used for normally distributed characteristics; median and interquartile range (IQR) are used for characteristics with a skewed distribution.

### Comparator Trials

We compared the baseline characteristics of participants enrolled in PROTECT to those of participants enrolled in other contemporary phase 3 trials with patients with IgAN. We identified the following 4 such studies: (i) Supportive Versus Immunosuppressive Therapy for the Treatment of Progressive IgA Nephropathy (STOP-IgAN; NCT00554502),^23^^,^^24^ (ii) Therapeutic Evaluation of Steroids in IgA Nephropathy Global Study (TESTING; NCT01560052) trial,^25^^,^^26^ (iii) Dapagliflozin and Prevention of Adverse Outcomes in CKD (DAPA-CKD; NCT03036150) trial,^27^^,^^28^ and (iv) Nefecon in Patients With Primary IgAN at Risk of Progressing to End-Stage Renal Disease (NefIgArd; NCT03643965).^29^^,^^30^ In contrast to the STOP-IGAN, TESTING, and NefIgArd trials, which are dedicated IgAN studies, the DAPA-CKD trial enrolled a broad CKD population and subgroup analyses were performed for those diagnosed with IgAN. For these studies, IgAN participants were compared with those recruited into PROTECT based on the eligibility criteria (Table 1). Although the Study of Heart and Kidney Protection with Empagliflozin (EMPA-KIDNEY; NCT03594110)^31^ includes 817 patients with IgAN among the total study population of 6609 patients with CKD, this study is not included in the comparisons because subgroup data specifically describing the characteristics of the patients with IgAN have not yet been provided.

**Table 1:** Comparison of key eligibility criteria for PROTECT and other contemporary phase 3 trials recruiting participants with IgAN

Parameter	PROTECT	STOP-IGAN^23^^,^^24^	TESTING^25^^,^^26^	DAPA-CKD^27^^,^^28^	NefIgArd^29^
Study drug	Sparsentan	Supportive care + Immunosuppression	Methylprednisolone	Dapagliflozin	Nefecon
Comparator	Irbesartan	Supportive care (ACEi/ARB/Statin)	Placebo	Placebo	Placebo
Patients with IgAN	404^a^	162^a^	503	270^a^	360
Age, years	≥18	≥18 to ≤70	≥14	≥18	≥18
Biopsy-proven IgAN requirement	Yes At any time in the past	Yes At any time in the past	Yes At any time in the past	No	Yes Within the past 10 yr
Proteinuria	Protein excretion ≥1 g/d	Protein excretion ≥0.75g/d to ≤3.5 g/d	Protein excretion ≥1 g/d	UA/C ≥200 mg/g to ≤5000 mg/g	Protein excretion ≥1 g/d or UP/C ≥0.8 g/g
eGFR or creatinine clearance, ml/min/1.73 m^2^	≥30	≥30 to <90	≥20 to ≤120	≥25 to ≤75	≥35 to ≤90
Systolic/diastolic blood pressure, mm Hg	≤150/100	No criterion preventing inclusion	≤160/110	No criterion	≤140/90
Optimized RAAS inhibition	Yes Stable MTD or MLD of ACEi and/or ARB at ≥50% MLD for ≥12 wks	Yes MTD or MLD of ACEi and/or ARB, achieved through dedicated 6-mo run-in period	Yes MTD of ACEi and/or ARB	Stable dose of ACEi and/or ARB for ≥4 wks	Yes Stable MTD or MLD of ACEi and/or ARB ≥3 mo

ACEi, angiotensin-converting enzyme inhibitor; ARB, angiotensin receptor blocker; eGFR, estimated glomerular filtration rate; IgAN, immunoglobulin A nephropathy; MLD, maximum labeled dose; MTD, maximum tolerated dose; RAAS, renin-angiotensin-aldosterone system inhibition; UA/C, urine albumin-to-creatinine ratio; UP/C, urine protein-to-creatinine ratio.

a Randomized and received study drug.

## Results

A total of 671 patients were screened; 406 patients from clinical sites in 18 countries met eligibility criteria and were enrolled and randomized into PROTECT. Two randomized patients withdrew from the study before initiating study treatment. The 404 randomized patients who received study drug were included in the primary analysis population, and their baseline characteristics are described here. Approximately half of the enrolled patients were from Europe (53%), followed by Asia Pacific (27%) and North America (20%). Patients had a median age of 46 years and were primarily male, White, and non-Hispanic; 28.5% of patients were Asian (Table 2). The initial kidney biopsy occurred a median of 4.0 years before enrollment. There was a documented history of hypertension in 76.5% of patients.

**Table 2:** Baseline demographic characteristics and relevant medical history of patients who were randomized and received study drug in PROTECT

Characteristic	All patients (*N* = 404)
Age at informed consent, yr	46.0 (37.0–56.0)
Sex	
Male	282 (69.8)
Female	122 (30.2)
Race^a^	
White	272 (67.3)
Asian	115 (28.5)
Black or African American	4 (1.0)
Other	13 (3.2)
Ethnicity	
Not Hispanic or Latino	368 (91.1)
Hispanic or Latino	33 (8.2)
Not reported	3 (0.7)
Age at IgAN diagnosis, yr^b^	38.5 (30.0–49.0)
Time from initial kidney biopsy to informed consent, yr^c^	4.0 (1.0–10.0)
History of diabetes and impaired fasting glucose^d^	43 (10.6)
History of hypertension	309 (76.5)
Blood pressure, mm Hg	
Systolic	129.0 ± 13.5
Diastolic	82.4 ± 10.6
BMI, kg/m^2^	28.4 ± 5.4

BMI, body mass index; IgAN, immunoglobulin A nephropathy; IQR, interquartile range.

Data are represented as n (%), median (IQR), or mean ± SD.

a Patients may have selected more than 1 race. “Other” race included American Indian or Alaskan Native, Native Hawaiian or Other Pacific Islander, and Other.

b Age at IgAN diagnosis is derived based on the year of IgAN diagnosis and year of birth.

c Time from initial biopsy is derived based on the year of the initial kidney biopsy and year of signed informed consent.

d Diabetes was not among the exclusionary criteria for the study; however, certain diabetes medications were prohibited during the double-blind period, including sodium-glucose cotransporter-2 inhibitors and thiazolidinediones.

### Laboratory Assessments

Baseline assessments showed median urine protein-to-creatinine ratio of 1.2 g/g, median urinary protein excretion of 1.8 g/d, and 12.1% of patients had nephrotic-range proteinuria (Table 3). Median urinary albumin excretion was 1493 mg/d, and median urine albumin-to-creatinine ratio was 1.1 g/g. The study population included patients with a broad range of eGFRs, with the largest portion in CKD stage 3B (35.1%; Table 3). Other laboratory tests showed values largely within the normal ranges (≥80% of patients within normal range), with exceptions of elevated total cholesterol in 41.3% of patients, elevated triglycerides in 46.0% of patients, and elevated serum creatinine and cystatin C in 79.5% and 80.2% of patients, respectively.

**Table 3:** Laboratory values at baseline in patients who were randomized and received study drug in PROTECT

Characteristic	All Patients (*N* = 404)
UP/C, g/g	1.2 (0.8–1.8)
Urinary protein excretion, g/d	1.8 (1.3–2.8)
Nephrotic-range proteinuria (>3.5 g/d)	49 (12.1)
UA/C, g/g	1.1 (0.7–1.5)
Urinary albumin excretion, mg/d	1493 (1073–2280)
eGFR^a^	
Mean ± SD	57.0 ± 24.0
Median (IQR)	50.0 (39.0–70.0)
eGFR	
≥90	51 (12.6)
≥60 to <90	97 (24.0)
≥45 to <60	94 (23.3)
≥30 to <45	142 (35.1)
≥15 to <30	20 (5.0)
Hemoglobin, g/l	138.7 ± 15.9
Plasma lipid profile, mmol/l	
Total cholesterol	5.0 ± 1.1
HDL cholesterol	1.3 ± 0.4
LDL cholesterol	2.8 ± 1.0
Triglycerides	1.9 ± 1.1
Serum albumin, g/l	
Mean ± SD	41.5 ± 3.8
Median (IQR)	42.0 (40.0–44.0)
Serum potassium, mmol/l	4.6 ± 0.4
Serum creatinine, μmol/l	136.1 ± 45.3
Serum cystatin C, mg/l	1.5 ± 0.4
Hematuria/microscopic hematuria^b^	225 (55.7)
ALT, U/l	21.8 ± 10.8
AST, U/l	21.1 ± 8.0

ALT, alanine transaminase; AST, aspartate transferase; eGFR, estimated glomerular filtration rate in ml/min per 1.73 m^2^; HDL, high-density lipoprotein; IQR, interquartile range; LDL, low-density lipoprotein; UA/C, urine albumin-to-creatinine ratio; UP/C, urine protein-to-creatinine ratio.

Data are given as n (%), median (IQR), or mean ± SD. A central laboratory was used for all laboratory testing analyses.

a eGFR was determined using the Chronic Kidney Disease Epidemiology formula.

b The assessment of microscopic hematuria was limited by the use of a central laboratory, resulting in an unreliable analysis because of the transport time and analysis delays.

### Blood Pressure and Medications: Kidney-Protective, Blood Pressure-Lowering, and Lipid-Lowering

**Figure 1:**
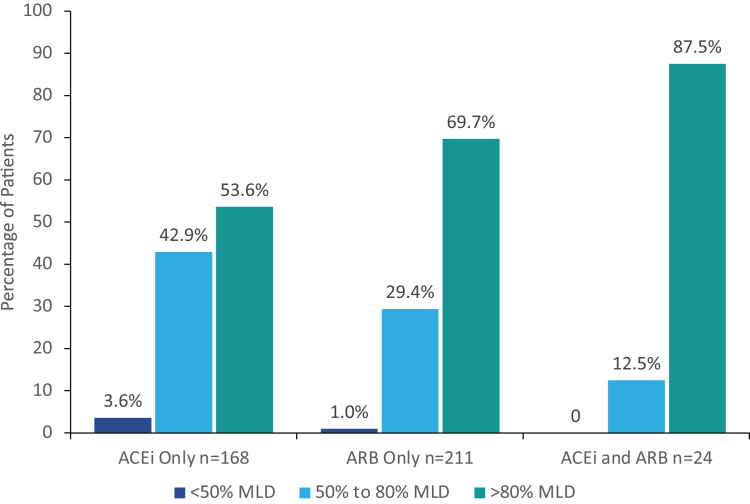
Patients receiving ACEi and ARB at screening at <50%, 50% to 80%, and >80% of MLD for patients on ACEi only, patients on ARB only, and patients on ACEi and ARB. For patients with more than 1 record of MLD percentage and for patients taking both ACEi and ARB treatment, the highest percentage MLD was included. ACEi, angiotensin-converting enzyme inhibitor; ARB, angiotensin receptor blocker; MLD, maximum labeled dose.

Before transitioning from ACEi and/or ARB treatment to study medication, mean systolic and diastolic blood pressure were 129.0 and 82.4 mm Hg, respectively. Of the 404 randomized patients that received study medication, 403 patients (99.8%) were receiving ACEi and/or ARB treatment at screening and during the screening period (Table 4). A higher percentage of patients were receiving ARB-only therapy (52.2%) versus ACEi-only therapy (41.6%). The MLD was received by 69.7% of patients receiving ARB only and 52.4% of patients receiving ACEi only. Among all patients, 63.4% (256/404) were receiving ACEi or ARB therapy at the MLD. The majority of patients (63.9%; 258/404) received ACEi or ARB doses at >80% of MLD and 97.8% (395/404) at doses ≥50% (Figure 1). Antihypertensive medications (besides RAASi) were being taken by 43.1% of patients, including diuretics by 15.4% of patients. Two or more antihypertensive medications (excluding RAASi) were taken by 15.1% of patients. The mean (SD) number of antihypertensive medications per patient (including RAASi medications before discontinuation) was 1.6 (0.8). Lipid-lowering medications were taken by 55.2% of patients.

**Table 4:** Medications at screening and baseline for patients who were randomized and received study drug in PROTECT

Characteristic	All Patients (*N* = 404)
ACEi and ARB treatment at screening^a^	
ACEi only, n (%, % on MLD)	168 (41.6, 52.4)
ARB only, n (%, % on MLD)	211 (52.2, 69.7)
ACEi and ARB, n (%, % on MLD of both, % on MLD of either)	24 (5.9, 37.5, 87.5)
Baseline medication use^b^	
Antihypertensive medications^c^	174 (43.1)
Diuretics^d^	62 (15.3)
Beta-blockers	55 (13.6)
Alpha-blockers	22 (5.4)
Calcium-channel blockers	109 (27.0)
Other	23 (5.7)
≥2 antihypertensive medications at baseline (excluding RAASi medications)	61 (15.1)
Number of antihypertensive medications per patient (including RAASi medications)	
Mean ± SD	1.6 ± 0.8
Median (IQR)	1.0 (1-2)
Lipid-lowering medications	223 (55.2)

ACEi, angiotensin-converting enzyme inhibitor; ARB, angiotensin receptor blocker; IQR, interquartile range; MLD, maximum labeled dose; RAASi, renin-angiotensin-aldosterone system inhibitors.

Data are given as n (%), unless otherwise indicated.

a ACEi and ARB treatment at screening; RAASi were prohibited during the study. Each “% on MLD” is based on the related n-value of patients receiving ACEi only, ARB only, or ACEi and ARB.

b Baseline medications were started before randomization (day 1) and continued after the initial dose of study medication.

c Antihypertensive medications exclude ACEis, ARBs, aldosterone blockers, and aliskiren.

d Diuretics include aldosterone antagonists (*n* = 2), thiazides (*n* = 18), and sulfonamides (*n* = 45).

### Asian Versus Non-Asian Geographic Regions

Considering the published differences in the clinical presentation and course of IgAN depending on site location globally, we further compared baseline characteristics in patients enrolled in Asian (including Hong Kong, Taiwan, and South Korea; *n* = 74) and non-Asian (all other countries; *n* = 330) geographic regions (Supplementary Tables S1−S3). These 2 populations were comparable by major characteristics, such as age, baseline eGFR, and proteinuria. However, there were a few notable numerical differences between the patients enrolled in these regions, which included a higher percentage of female patients, lower blood pressure, lower mean cholesterol, lower proportion of patients with high cholesterol, and lower proportion of patients with history of hypertension and baseline antihypertensive treatment in the Asian countries. Moreover, a higher percentage of patients from Asian geographic regions were receiving lipid-lowering medication. The percentage of patients receiving MLD of ACEi or ARB at baseline was higher in patients from non-Asian than in those from Asian regions (67.0% vs. 47.3%, respectively). In addition, there were patients of Asian race enrolled in non-Asian geographic regions. Similar to patients from Asian countries, this subpopulation had a lower proportion of males and history of hypertension compared with non-Asian patients (Supplementary Tables S4−S6; Figure S1).

### Comparison of PROTECT and Contemporary Phase 3 IgAN Trials

Selected baseline characteristics of patients in recent phase 3 IgAN studies are shown in Table 5. An average or median age of 40 to 50 years for patients at baseline was comparable across PROTECT, STOP-IGAN, and NefIgArd, whereas the median age was lower for TESTING (approximately 36 years), likely reflecting a short median duration from time of biopsy to enrollment (i.e., 5 months vs. 48 months for PROTECT).^24^^,^^26^^,^^27^^,^^29^ The average age of patients with IgAN participating in DAPA-CKD was older (51 years), potentially a consequence of an eGFR inclusion criterion orientating the study to more progressed patients.^27^ The mean (SD) eGFR of DAPA-CKD patients with IgAN was 43.8 (12.2) ml/min per 1.73 m^2^, in contrast to the other studies which had mean or median eGFR ranging between 50 and 60 ml/min per 1.73 m^2^.^24^^,^^26^^,^^27^^,^^29^ The geographic enrollment of patients varied considerably between studies with STOP-IGAN recruiting solely from centers in Germany,^24^ with TESTING recruiting 95% Asian patients,^26^ and NefIgArd recruiting 85.9% White patients.^29^ PROTECT and DAPA-CKD patients with IgAN had more balance between Asian and White patients (PROTECT, 28% and 67%, respectively; DAPA-CKD, 59% and 40%, respectively).^27^ None of these studies reported greater than 5% non-Asian and non-White populations.

**Table 5:** Select baseline characteristics for PROTECT and other contemporary phase 3 trials with participants with IgAN

Characteristic	PROTECT	STOP-IgAN^23^^,^^24^		TESTING^25^^,^^26^		DAPA-CKD IgAN Patients^27^^,^^28^		NefIgArd^29^^,^^30^^,^^a^	
	Total (*N* = 404)	SC (*n* = 80)	SC + IST (*n* = 82)	Methylprednisolone (*n* = 257)	Placebo (*n* = 246)	DAPA (*n* = 137)	Placebo (*n* = 133)	Nefecon (*n* = 97)	Placebo (*n* = 102)
Age, years, mean ± SD or median (IQR)	46.0 (37.0–56.0)	45.8 ± 12.5	42.8 ± 13.1	35.6 (29.4–46.3)	36.6 (29.0–45.9)	52.2 ± 13.1	50.1 ± 13.1	44 (range, 25–69)	43 (range, 23–73)
Sex, %									
Male	69.8	81	76	60	61	67.9	66.9	70.1	65.7
Female	30.2	19	24	40	39	32.1	33.1	29.9	34.3
Race, %									
White	67.3	NR	100^b^	5	5	39.4	40.6	87.6	84.3
Asian	28.5	NR	0	95	95	59.9	57.9	11.3	12.7
Black or African American	1.0	NR	0	0	0	0	0.8	0	0
Other	3.2	NR	0	0	0	0.7	0.8	1.0	2.9
Systolic BP, mm Hg, mean ± SD or median (IQR)	129.0 ± 13.5	127 ± 8.5	124 ± 9.7	123.8 (115.0–132.5)	125.0 (115.5–131.0)	127.7 ± 16.2	127.0 ± 13.9	128 (122–134)	124 (117–131)
Diastolic BP, mm Hg, mean ± SD or median (IQR)	82.4 ± 10.6	78 ± 7.0	77 ± 7.0	80.0 (73.5–85.0)	80.0 (74.0–86.0)	78.7 ± 11.8	79.5 ± 10.1	79 (76–84)	78 (73–83)
BMI, kg/m^2^, mean ± SD or median (IQR)	28.4 ± 5.4	28.6 ± 5.3	27.0 ± 5.0	24.2 (21.6–26.7)	24.7 (22.0–28.0)	26.3 ± 4.2	27.6 ± 6.1	29 (26–32)	28 (24–31)
eGFR									
Mean ± SD	57.0 ± 24.0	57.4 ± 24.9	61.1 ± 29.0	NR	NR	44.3 ± 12.4	43.2 ± 12.0	NR	NR
Median (IQR)	50.0 (39.0–70.0)	NR	NR	56.1 (43.2–75.0)	59.0 (42.0–77.6)	NR	NR	54.9 (46.4–68.9)	55.5 (45.5–67.7)
Total protein excretion, g/d mean ± SD or median (IQR)	1.8 (1.3–2.8)	1.6 ± 0.7	1.8 ± 0.8	1.99 (1.36–3.09)	1.93 (1.38–2.88)	NR	NR	2.3 (1.7–3.3)	2.3 (1.5–3.6)
UP/C, g/g, mean ± SD or median (IQR)	1.2 (0.8–1.8)	1.0 ± 0.5	1.1 ± 0.6	NR	NR	NR	NR	1.3 (1.0–1.8)	1.2 (0.9–1.8)
UA/C, g/g, median (IQR)	1.1 (0.7–1.5)	NR	NR	NR	NR	0.9 (0.6–1.5)	0.9 (0.5–1.6)	1.0 (0.8–1.4)	1.0 (0.7–1.6)
RAASi, %^c^									
ACEi	41.6	34	49	54.5	52.0	32.1	30.8	55.7	43.1
ARB	52.2	30	15	46.3	48.8	65.0	72.2	39.2	47.1
ACEi and ARB	5.9	32	36	NR	NR	NR	NR	3.1	6.9
No RAASi	0.2	4	0	0	0	0	0	2.1	2.9
% MLD ACEi/ARB, %									
<50% MLD	2.0	NR	NR	11.7	14.2	NR	NR	22.7	19.6
≥50% MLD	97.8	NR	NR	86.4	81.7	NR	NR	75.3	79.4
≥50% to 80% MLD	34.0	NR	NR	NR	NR	NR	NR	22.7^d^	32.4^d^
>80% MLD	63.9	NR	NR	NR	NR	NR	NR	52.6^d^	47.1^d^
100% MLD	63.4	76.0	71.0	NR	NR	NR	NR	NR	NR
≥1 non-RAASi anti-hypertensives, %	43.1	NR	NR	NR	NR	NR	NR	NR	NR
Diuretics	15.4	NR	NR	7.8	8.5	21.2	27.1	16.5	17.6
Calcium-channel blockers	27.0	NR	NR	22.2	23.6	NR	NR	38.1	34.3
Lipid-lowering medication, %	55.2	73	81	NR	NR	49.6	50.4	23.7	13.7
Kidney biopsy, %	100 (IC)	100 (IC)		100 (IC)		94.2 (not an IC)	94.0 (not an IC)	100 (IC)	
Time since kidney biopsy, mo, median (IQR)	48 (12–120)	NR	NR	5 (4–11)	5 (3–14)	NR	NR	24 (9.6–73.2)	33.6 (6.0–85.2)

ACEi, angiotensin-converting enzyme inhibitor; ARB, angiotensin receptor blocker; DAPA, dapagliflozin; eGFR, estimated glomerular filtration rate in ml/min per 1.73 m^2^; IC, inclusion criterion; IQR, interquartile range; IST, immunosuppression treatment; MLD, maximum labeled dose; NR, not reported; RAASi, renin-angiotensin-aldosterone system inhibitors; SC, supportive care with RAS inhibition; UA/C, urine albumin-to-creatinine ratio; UP/C, urine protein-to-creatinine ratio.

a Total recruited patients, *N* = 360. Total patients included in the efficacy analyses of part A, *N* = 199.

b Personal communication from Jurgen Floege, August 2022.

c RAASi therapy at baseline was continued during the study in all patient groups in each study except for the PROTECT study.

d MLD categories reported as ≥50% and <80% MLD and ≥80% MLD.

Proteinuria at baseline was very similar between the IgAN-dedicated studies with mean or median protein excretion levels of approximately 1.6 to 2.0 g/d or a mean or median urine protein-to-creatinine ratio of 1.0 to 1.3 g/g.^24^^,^^26^^,^^29^ Albuminuria was also measured in PROTECT with a median urine albumin-to-creatinine ratio of 1.1 g/g (IQR, 0.7−1.5 g/g), which was slightly greater than the median urine albumin-to-creatinine ratio of 0.9 g/g (IQR, 0.5−1.5 g/g) for DAPA-CKD patients with IgAN.^27^ Systolic and diastolic blood pressure were comparable across studies ranging from a mean or median systolic blood pressure of 124 to 129 mm Hg and a mean or median diastolic blood pressure of 77 to 82 mm Hg, with a tendency for lower systolic blood pressure levels in TESTING and STOP-IGAN.^24^^,^^26^^,^^27^^,^^29^ In all these studies, close to 100% of patients were on an ACEi and/or ARB at screening or baseline. STOP-IGAN had over 30% of patients on dual RAASi, and this was substantially lower in PROTECT at 5.9%.^24^ The percentage of patients receiving 100% of the MLD of an ACEi and/or ARB was highest in STOP-IGAN (76.0% in the supportive care group and 71.0% in the immunosuppression group) and PROTECT (63.4%), whereas in NefIgArd, 49.7% of patients received ≥80% of the MLD.^24^^,^^29^ In TESTING, ACEi and/or ARB at ≥50% of MLD was received by 84.1% of patients (86.4% receiving methylprednisolone and 81.7% receiving placebo), as well as in 77.4% of NefIgArd patients (75.3% receiving nefecon and 79.4% receiving placebo), and in 97.8% of PROTECT patients.^26^^,^^29^ The percentage of patients receiving MLD or a percentage of MLD was not reported in DAPA-CKD.^27^ Diuretics were most commonly used in DAPA-CKD patients with IgAN (24.1%) compared to PROTECT (15.4%), TESTING (approximately 8%), and NefIgArd (approximately 17%).^24^^,^^26^^,^^27^^,^^29^ Approximately 75% of patients in STOP-IGAN were on lipid-lowering medications at baseline compared to 55% for PROTECT, 50% for DAPA-CKD patients with IgAN, and 19% for NefIgArd.^24^^,^^27^^,^^29^

## Discussion

The PROTECT trial is evaluating sparsentan, a novel nonimmunosuppressive single-molecule dual endothelin angiotensin receptor antagonist with angiotensin II subtype 1 receptor and endothelin type A receptor antagonist-associated hemodynamic, anti-inflammatory, antifibrotic, and podocyte-protective effects in patients with IgAN.^11^^,^^13^^,^^17^ PROTECT is one of the largest interventional trials testing a novel drug versus standard of care in IgAN to date. In recent years, several phase 3 studies have been initiated in IgAN. These contemporary trials tend to align in enrolling patients at high risk of progression, typically defined as proteinuria ≥1 g/d despite ≥3 months of stable RAASi at a maximum tolerated or labeled dose.^24^^,^^26^^,^^27^^,^^29^^,^^30^ Indeed, these basic eligibility parameters are consistent with the recently published KDIGO guidelines, which recommend consideration of patients for a clinical trial if proteinuria remains above 0.75 to 1.0 g/d despite 3 months of maximized RAASi.^10^ The phase 3 trials PROTECT, STOP-IGAN, TESTING, and NefIgArd all abide by these features, and despite recruiting patients from different geographies, proteinuria levels at study entry are comparable.^24^^,^^26^^,^^27^^,^^29^^,^^30^ The extent of RAASi at MLD can be compared between PROTECT and STOP-IGAN, noting that STOP-IGAN applied a 6-month run-in phase to optimize supportive care, including RAASis. In PROTECT, 63.4% of patients were on the MLD of an ACEi and/or ARB at screening, whereas this was achieved in approximately 73% of patients in STOP-IGAN.^24^ When considering the extent of RAASis at ≥50% of MLD among PROTECT, TESTING, and NefIgArd participants, the percentage of patients is highest in PROTECT (97.8% vs. 77.4% to 84.1%).^26^^,^^30^

In contrast to these dedicated IgAN studies, phase 3 trials enrolling a broad spectrum of patients with diabetic and nondiabetic CKD have been initiated. These studies, such as DAPA-CKD,^32^ which is testing the SGLT2 inhibitor dapagliflozin, include patients with IgAN; however, the requirement for maximizing RAASi tends to be less stringent (e.g., requirement for stable dose for at least 4 weeks, not specifically stating a requirement for an optimized dose or an MLD, and only if ACEi and/or ARB are not medically contraindicated^27^), and lower proteinuria thresholds equivalent to <1 g/d are applied. Yet, the comparison of PROTECT with this trial deserves special attention, because both trials tested a nonimmunosuppressive intervention. The patients with IgAN enrolled in DAPA-CKD had lower albuminuria at baseline compared to PROTECT and substantially lower mean eGFR (44 vs. 57 ml/min per 1.73 m^2^).^27^ In DAPA-CKD, the lower baseline eGFR, as a consequence of an eGFR inclusion criterion of 25 to 75 ml/min per 1.73 m^2^, shifted the target population to those with more progressed disease. Low eGFR is a strong predictor of kidney failure,^33^ which may explain the high rate of kidney failure events in DAPA-CKD patients with IgAN. In contrast to DAPA-CKD, PROTECT allowed for inclusion of patients across CKD stages 1 to 3B, with reasonable balance of enrolled patients across these stages. Furthermore, although study entry criteria required an eGFR ≥30 ml/min per 1.73 m^2^ at screening, 20 patients (5%) experienced eGFR <30 ml/min per 1.73 m^2^ at baseline (minimum eGFR was 24 ml/min per 1.73 m^2^). The benefit of this broad approach will be the ability to evaluate the treatment effects of sparsentan versus irbesartan from early to later stages of disease progression.

As noted previously, phase 3 studies have varied greatly in the geographic region in which they were conducted, which may be reflected in some of the differences in certain patient baseline characteristics. PROTECT, DAPA-CKD, and NefIgArd are global studies enrolling patients across multiple continents. In contrast, STOP-IGAN recruited patients from 32 centers in Germany and TESTING was predominantly in sites in China.^24^^,^^26^^,^^27^^,^^29^^,^^30^ As examples, body mass index is lower in TESTING patients, whereas the ratio of males to females (60:40) is more balanced than in STOP-IGAN (78:22), with the other trials falling between these 2 studies. Differences at baseline noted between patients in PROTECT enrolled from Asian and non-Asian geographic regions included a higher percentage of female patients in Asian than in non-Asian regions, and a higher percentage of patients with a history of hypertension and baseline antihypertensive medication use in non-Asian regions. The percentage of patients receiving MLD of ACEi or ARB at screening was also higher in patients from non-Asian regions. However, the key measures of kidney function, proteinuria, and eGFR were similar in Asian versus non-Asian patients. In the final analysis, enrollment of a large diverse and representative population like in PROTECT will be an important consideration allowing for a comprehensive evaluation of the therapeutic response to sparsentan in patients with IgAN regardless of region and ethnicity/race.^34^

A final consideration when comparing the phase 3 IgAN trials is the choice of comparator. Unlike the other phase 3 studies and other ongoing phase 2 and phase 3 trials in IgAN, PROTECT is unique in the double-blind randomized application of an active control, irbesartan, to compare to the investigational drug, sparsentan. Each of the IgAN-dedicated studies relies on the investigators to use their best judgment as to whether a patient is on a maximum tolerated dose if the patient is on less than the MLD of an unblinded RAASi at study entry.^24^^,^^26^^,^^29^^,^^30^ However, applying a double-blind randomization scheme to standardize the up-titration of the standard-of-care RAASi comparator, the procedure followed in PROTECT, introduces another layer of rigor to the study when assessing the efficacy of the investigational drug.

A limitation of the PROTECT trial is that the assessment of microscopic hematuria was not possible given the use of a central laboratory for analyses. This would have resulted in an unreliable analysis of microhematuria because of the transport time and analysis delays. In addition, there was no systematic collection of history of macroscopic hematuria from patients at study entry. The MLD of ACEis and ARBs may be defined differently in different geographic regions. To minimize heterogeneity for assessment of eligibility and reporting, a strength of the PROTECT trial is the use of a standardized daily doses table that was applied on the basis of a consensus of the majority of participating countries (Supplementary Table S7).

### Conclusion

Sparsentan is a novel single-molecule dual endothelin angiotensin receptor antagonist that is not an immunosuppressant agent. It is being evaluated in PROTECT as a promising new therapeutic in IgAN, an entity with unmet need for novel therapies. The patients enrolled in PROTECT were adults with biopsy-confirmed IgAN (excluding IgAN secondary to another condition or IgA vasculitis) and at high risk of progression to kidney failure requiring dialysis or kidney transplantation based on urinary protein excretion ≥1.0 g/d despite maximum standard-of-care treatment with ACEi and ARB at screening. The enrollment of patients across both (i) Asian and non-Asian geographic regions with differing racial backgrounds, and (ii) a full range of CKD stages will enable characterization of the treatment effect of sparsentan in high-risk patients with IgAN across geographic regions and levels of kidney function, respectively.

## Appendix

### List of PROTECT Investigators

#### Australia

Bhadran Bose, Muralikrishna Gangadharan, Stephen McDonald, Chen Peh, Sadia Jahan, Chii Yeap, Philip Clayton, Georgina Irish, Nikhil Thyagarajan, Peter Hollett, Rathika Krishnasamy, Robert Carroll, Shilpanjali Jesudason, Susan Crail, Toby Coates, Jane Waugh, Euan Noble, Kumaradevan Mahadevan, Victoria Campbell, Tania Salehi, Wai Lim, Neil Boudville, Aron Chakera, Doris Chan, Anoushka Krishnan, Yusuf Eqbal, Alastair Gillies, Eswari Vilayur, Thida Maung Maung Myint, Nicholas Gray, Jane Waugh, Euan Noble, Melissa Cheetham, Yusuf Eqbal, Peter Hollett, Rathika Krishnasamy, Kumaradevan Mahadevan, Victoria Campbell, Carol Pollock, Bruce Cooper, Amanda Mather, Sarah Roxburgh, Yvonne Shen, Stefanie Stangenberg, Amanda Siriwardana, Muh Geot Wong, Emma O’Lone, Susan Wan, Brendon Neuen, Jeffrey Tsun Kit Ha, Dana Kim, Lauren Heath, Arunima Jain, Elaine Phua, Yan Li, Martin Gallagher, Meg Jardine, Angus Ritchie, Mona Razavian, Celine Foote, Roger Wyndham, Shaundeep Sen, Zoltan Endre, Jonathan Erlich, Mangalee Fernando, Kenneth Yong, Grant Luxton, Sradha Kotwal, Simon Roger, Vidu Wijeratne, David Packham, and Ian Fraser.

#### Belgium

Bert Vandewiele, Margo Laute, Wim Lemahieu, Sofie Jamar, Sara Ombelet, Gert Meeus, Marc Decupere, Olivier Schockaert, Peter Doubel, Liesbeth Viaene, Luc Radermacher, Catherine Masset, Martial Moonen, Eric Firre, Martina Milicevic, Xavier Warling, Bart Maes, An Vanacker, and Thomas Malfait.

#### Croatia

Ivan Durlen, Ivica Horvatic, Ana Savuk, Lana Gellineo, Sandra Karanovic, Zivka Dika, Bojan Jelakovic, Djuro Plavljanic, Ivana Mikacic, Dubravka Trajbar Kentric, Dunja Barisic, Marija Stankovic, Karolina Majstorovic Barac, Ivan Kruljac, Drasko Pavlovic, Martin Drinkovic, Ingrid Prkacin, Jerko Barbic, Zvonimir Sitas, and Dunja Vujcic.

#### Czech Republic

Ivan Rychlik, Anna Benesova, Klara Drinovska, Karolina Kratka, Vladimir Tesar, and Dita Maixnerova.

#### Estonia

Madis Ilmoja, Kristin Unt, Kadri Lilienthal, Asta Auerbach, Liisi Leis, Julia Piel, Annika Adoberg, Mai Rosenberg, Kulli Kolvald, Kristi Veermae, Kadri Telling, Elviira Seppet, and Jana Uhlinova.

#### France

Philippe Zaoui, Pierre-Louis Carron, Ingrid Masson, Miriana Dinic, Damien Thibaudin, Christian Broyet, Nicolas Maillard, Hesham Mohey, Christophe Mariat, Guillaume Claisse, Eric Alamartine, Bertrand Dussol, Stephane Burtey, Noemie Chiche-Jourde, Jean-Emmanuel Serre, Guillaume Jeantet, Leila Chenine, Anne Blanchard, Stephane Roueff, Eric Thervet, David Fouassier, Alexandre Buffet, Marine Livrozet, Roxane Gaisset, Alexandre Karras, Anne-Elisabeth Heng, Cyril Garrouste, Carole Philipponnet, Clementine Nicolo, Alba Atenza, Camille Lanaret, Clarisse Greze, Valentin Mayet, Clement Dumond, Yahsou Delmas, Christian Combe, Claire Rigothier, Laure Burguet, Aurore Labat, Simon Mucha, and Valérie de Précigout.

#### Germany

Thomas Weinreich, Helmut Reichel, Diliana Draganova, Lothar Wolf, Bernd Hohenstein, Sven Heinrichs, Simone Kulka, Sebahat Sat, Lea Weiland, Thilo Krueger, Gunter Wolf, Christiane Kettner, Mandy Schlosser, Johann Konstantin Herfurth, Annegret Koch, Martin Busch, Stephan Christian Werth, Martin Nitschke, Figen Cakiroglu, Franziska Sarnow, Lisa Schulz, Stefan Weiner, Nikolaus Wirtz, Eric Koester, Marcus Moeller, Juergen Floege, Eleni Stamellou, Silja Sanden, Hans Schmidt-Guertler, Wanja Bernhardt, Margret Patecki, Georg Schlieper, Kevin Schulte, Annette Girardet, and Ulrich Kunzendorf.

#### Hong Kong

Sydney Chi Wai Tang, Lorraine Pui Yuen Kwan, Maggie Ming Yee Mok, Gary Chi Wang Chan, Mingyao Ma, Davina Ngoi Wah Lie, Anthony Ting Pong Chan, Cheuk Chun Szeto, Kit Chung Jack Ng, Siu Fai Cheung, Tak Tai Andrew Yue, Ka Shun Samuel Fung, Hon Tang, Ka Fai Yim, Wai Ping Law, Yick Hei Wong, Chi Kwan Darwin Lam, and Sze Ho Sunny Wong.

#### Italy

Carmelita Marcantoni, Roberta Aliotta, Francesca Deodato, Gemma Patella, Nicolino Comi, Caterina Vita, Nazareno Carullo, Davide Bolignano, Michela Musolino, Matias Trillini, Norberto Perico, Giuseppe Remuzzi, Erica Daina, Luigi Biancone, Loredana Colla, Manuel Burdese, Chiara Cogno, Elena Boaglio, Isabella Abbasciano, Carlotta Federica Zizzi, Paolo Randone, Pietro Napodano, Anna Ricchiuto, Matthias Cassia, Simone Accarino, Mario Cozzolino, Rocco Baccaro, Stefano Costanzi, Federica Di Maio, Maria Arena, Federica Urciuolo, Sara Vigano, Andrea Cavalli, Monica Limardo, Monica Bordoli, Serena Ponti, Selena Longhi, Andrea Solazzo, Francesco Giaroni, Gabriele Donati, Massimo Torreggiani, Davide Catucci, Marco Colucci, Vittoria Esposito, Ciro Esposito, Loreto Gesualdo, Flavia Capaccio, Emma Diletta Stea, Carmen Sivo, Francesca Annese, Federica Papadia, Mirco Belingheri, Patrizia Passerini, Silvia Malvica, and Piergiorgio Messa.

#### Lithuania

Marius Miglinas, Alvita Vickiene, Urte Zakauskiene, Egle Asakiene, Inga Arune Bumblyte', Asta Stankuviene, and Lina Santockiene.

#### New Zealand

Ashik Hayat, Allister Williams, Peter Sizeland, Kannaiyan Rabindranath, Eddie Tan, Gerald Waters, Lai Wan Chan, Andrew Henderson, Angus Turnbull, Andrew McNally, Annie Reynolds, Helen Pilmore, Ian Dittmer, Paul Manley, Elizabeth Stallworthy, Tze Goh, David Semple, Michael Collins, Elizabeth Curry, Jafar Ahmed, and Thu Nguyen.

#### Poland

Agata Winiarska, Justyna Zbrzezniak, Tomasz Stompor, Magdalena Krajewska, Hanna Augustyniak-Bartosik, Dorota Zielinska, Anna Jander, Malgorzata Stanczyk, Marcin Tkaczyk, Przemyslaw Miarka, Dariusz Aksamit, Piotr Jaskowski, Wladyslaw Sulowicz, Dominik Cieniawski, Julita Gontarek-Kacprzak, Robert Malecki, Elzbieta Felicjanczuk, Norbert Kwella, Bogna Kwella, and Ewa Satora.

#### Portugal

João Carlos Fernandes, Ana Marta Gomes, Marina Reis, Daniela Lopes, Catarina Almeida, Helena Sá, Ana Carolina Figueiredo, Clara Pardinhas, Edgar Almeida, Mario Raimundo, Ana Cortesão Costa, Luis Pedro Falcao Goncalves, Sara Fernandes, Sónia Silva, Catarina Teixeira, Adriana Fernandes, Fernando Nolasco, Patricia Alves, Mario Gois, Nuno Fonseca, Ana Messias, Maria Menezes, Filipa Cardoso, Helena Sousa, Joana Marques, Rui Barata, Jose Antonio Lopes, Sofia Jorge, Joana Gameiro, Jose Nuno de Almeida Agapito Fonseca, Sara Goncalves, Ana Farinha, Patricia Valerio Santos, Ana Natario, Jose Carlos de Jesus Barreto, Catarina Abrantes, Elsa Sofia Quadrado Soares, Joana de Sousa Soares Felgueiras, Liliana Cunha, Lucia Parreira, Teresa Furtado, and Alvaro Vaz.

#### South Korea

Kook-Hwan Oh, Hajeong Lee, Se Joong Kim, Dong-Wan Chae, Jong Cheol Jeong, Yeong Hoon Kim, Yunmi Kim, Hyeong Cheon Park, Hoon Young Choi, Hyung Wook Kim, Moon Hyoung Lee, Songuk Yoon, Kyu-Beck Lee, Young Youl Hyun, Tae-Hyun Yoo, Seung Hyeok Han, Jung Tak Park, Sunggyun Kim, Young Rim Song, Jwa-Kyung Kim, Hyung-seok Lee, Narae Joo, JungEun Lee, Hye Ryoun Jang, Junseok Jeon, Wookyung Chung, Hyun Hee Lee, Jae Hyun Chang, Ka Yeong Chun, JiYong Jung, Han Ro, Aejin Kim, Sang-Kyung Jo, Jihyun Yang, Myung-Gyu Kim, and SeWon Oh.

#### Spain

Caridad Martinez Villanueva, Ana Vilar Gimeno, Gustavo Andres Useche Bonilla, Esther Tamarit, Antonio Galan Serrano, Eduardo Verde Moreno, Jose Luño Fernandez, Maria Angeles Goicoechea Diezhandino, Ursula Verdalles Guzman, Ana Perez de Jose, Alberto Ortiz Arduan, María Vanessa Pérez Gómez, Catalina Martín Cleary, Raul Fernandez Prado, Elena Goma, Jose Ballarin, Montserrat Diaz Encarnacion, Iara Da Silva Santos, Helena Marco Rusinol, Monica Furlano, Carlos Arias, Clara Barrios, Eva Rodriguez Garcia, Adriana Sierra Ochoa, Belen Vizcaino Castillo, Jonay Pantoja Perez, Mercedes Gonzalez Moya, Mari Sargsyan, Emma Calatayud Aristoy, Ana Avila Bernabeu, Leticia Perez Lluna, Tamara Malek Marin, Maria Antonia Munar Vila, Ivon Maritza Bobadilla Rico, Natalia Allende Burgos, Eduardo Gutierrez Martinez, Elena Gutierrez Solis, Angel Sevillano, Evangelina Merida Herrero, Josep Miquel Blasco Pelicano, Lida Maria Rodas Marin, Luis F Quintana, Maria Antonieta Azancot Rivero, Natalia Ramos Terrades, Clara Garcia Carro, Irene Agraz Pamplona, Mercedes Salgueira Lazo, Francisco de la Prada Alvarez, Fabiola Alonso Garcia, Wenceslao Adrian Aguilera Morales, Salia Virxinia Pol Heres, Angel Forcen, Eduardo Parra Moncasi, Cristina Medrano Villarroya, Alejandro Soria Villen, Olga Gracia Garcia, Mercedes Velo Plaza, Maria Dolores Sánchez de la Nieta, Marta Calvo Arevalo, Antolina Moreno, Secundino Cigarran Guldris, Manuel Pereira de Vicente, Maria Antonia Munar Vila, Ivon Maritza Bobadilla Rico, and Natalia Allende Burgos

#### Taiwan

Bang-Gee Hsu, Chih-Hsien Wang, Cheng-Hsu Chen, Tung-Min Yu, Ming-Ju Wu, Shang-Feng Tsai, Chia-Tien Hsu, Hsien-Fu Chiu, Kang-Ju Chou, Hua-Chang Fang, Po-Tsang Lee, Hsin-Yu Chen, Chien-Liang Chen, Chien-Wei Huang, Shih-Hsiang Ou, Tzung-Yo Ho, Chih-Yang Hsu, Ming-Shan Chang, Yen-Ling Chiu, Yu-Sen Peng, Kai-Hsiang Shu, Szu-Yu Pan, Shih-Ping Hsu, Ju-Yeh Yang, Mei-Fen Pai, Po-Yu Tseng, Hon-Yen Wu, Wan-Chuan Tsai, Kuei-Ting Tung, Hung-Yuan Chen, Hung-Chun Chen, Shang-Jyh Hwang, Mei-Chuan Kuo, Daw-Yang Hwang, Yi-Wen Chiu, Chi-Chih Hung, Hung-Tien Kuo, and Jer-Chia Tsai.

#### United Kingdom

Kieran McCafferty, Suzanne Forbes, Indranil Dasgupta, Mark Thomas, Amar Mahdi, Bamidele Ajayi, Paramit Chowdhury, Theodoros Kasimatis, Dimitrios Moutzouris, Caroline Dudreuilh, Rishi Pruthi, Nick Mansfield, Gabriel Doctor, Sapna Shah, Sui Kon, Priscilla Smith, Patrick Hamilton, Durga Kanigicherla, Omar Sherin Ibrahim Ragy, Bassam Alchi, Oliver Flossmann, Farid Ghalli, Sarah Lawman, Smeeta Sinha, Constantina Chrysochou, Chukwuma Chukwu, Aine Maire De Bhailis, Saif Al Chalabi, Amy Hudson, Arun Gopu, Olivia Wickens, Joshua Storrar, Mona Wahba, Nathan Lorde, Mohammad Rony, Sian Griffin, Farah Latif, Mohammad Ali, Louise DaSilva, Jonathan Ayling-Smith, Eamon Mahdi, Lisa Willcocks, Rachel Jones, Jonathan Barratt, Chee Kay Cheung, Haresh Selvaskandan, Dan Pugh, Matthew Sayer, Neeraj Dhaun, Fiona Chapman, Patrick Mark, Colin Geddes, Emily McQuarrie, Rajan Patel, Laurence Solomon, Arvind Ponnusamy, Adam Morris, Pedro Okoh, Lauren Floyd, Ajay Dhaygude, Janson Leung, Christopher Goldsmith, Bhavna Pandya, Didem Tez, Ashraf Mikhail, Karen Brown, Thomas Bucknall, and Mark Lambie.

#### United States

Roderick Comunale, Donald Brandon, Stacy Martinez, Amanda Hall, Amy Henderson, Aaron Fearday, Nicole Douthit, Brian Snow, Arnold Silva, Cathylee Sly, Christopher Keller, Robert Davidson, Jerry Meng, Robert Haws, Siddhartha Kattamanchi, Javad Mojarrab, Unnikrishnan Pillai, Richard Lafayette, Michelle O'Shaughnessy, Fahameedah Kamal, Kshama Mehta, Bruce Baker, Mario Ruiz, Praveena Jyothinagaram, Usha Peri, William Paxton, James Tumlin, Kerri McGreal, Ellen McCarthy, Cassandra Kimber, Archana Gautam, Kassem Khalil, Viet Nguyen, Viet Nguyen, Raffi Minasian, Dariush Arfaania, Sam Daneshvari, Michel Zakari, Artashes Patrikyan, Rouzbeh Afsari, Christine Ayvazyan, Faisal Fakih, Mark Lagatta, Faisal Fakih, Alfred Rodriguez, Jorge Enrique Monroy Avella, Ramachandra Patak, Jigar Kadakia, Jai Radhakrishnan, Gerald Appel, Wooin Ahn, Bradley Nelson, Allyson Medina, Syeda Ahmad, Yonatan Peleg, Nisha Clement, Ian Chiu, Elizabeth Hendren, Andrew Bomback, Pietro Canetta, Bruce Spinowitz, Chaim Charytan, Nishita Parikh, Sheng Kuo, Ritesh Raichoudhury, Mirela Dobre, Lavinia Negrea, Aparna Padiyar, Arksarapuk Jittirat, Nishigandha Pradhan, Ranjit Dhelaria, Saravanan Balamuthusamy, Machaiah Madhrira, Thomas Powell, Howard Lifland, Asha Bailey, Sarah Ashley Ford Sightler, Meera Patel Suthar, Heather Green, Samir Parikh, Isabelle Ayoub, Brad Rovin, Salem Almaani, Gabriel Contreras, Alessia Fornoni, Yelena Drexler, Abdallah Geara, Brittany Sheridan, Gaia Coppock, Jonathan Hogan, Carlos Gonzalez, Shamik Bhadra, Pradip Chowdhury, Kay Kyaw, May Tan, Lathika Raakesh, Elder Mendoza, Veronica Viramontes, Asghar Chaudhry, Juan Carbonell, Rajdeep Gadh, Victor Fernandez, Mohamad Kassem, Radu Jacob, Karen Wilder, Britt Newsome, Kathryn Klamm, Irina Suyumova, Laura Ann Kooienga, Catherine Janko, Dana Rizk, Bruce Julian, Dawn Caster, Erika Perez, Gunjan Garg, Nayan Gowda, Suneel Udani, Sreedhar Mandayam, Biruh Workeneh, Roderick Comunale, Donald Brandon, Unnikrishnan Pillai, Ali Assefi, Barbara Greco, Michael Germain, Jusmin Patel, Sarah Quinn, James Sullivan, Jeffrey Glaze, Phillip Madonia, Kellyn McMahon, Harold Giles, Sharon Adler, and Tiane Dai.

## Disclosure

JB has received research grants from Argenx, Calliditas Therapeutics, Chinook Therapeutics, Galapagos NV, GlaxoSmithKline, Novartis, and Travere Therapeutics Inc.; and is a medical/scientific advisor to Alnylam Pharmaceuticals, Argenx, Astellas Pharma, BioCryst Pharmaceuticals, Calliditas Therapeutics, Chinook Therapeutics, Dimerix, Galapagos NV, GlaxoSmithKline, Novartis, Travere Therapeutics Inc., UCB, Vera Therapeutics, and Visterra. BR is a consultant to Calliditas, Novartis, Omeros, Travere Therapeutics Inc., and Vera. MGW received honorarium from Alpine, Amgen, AstraZeneca, Baxter, Chinook, CSL Behring, Dimerix, Eledon, George Clinical, Horizon, Otsuka, and Travere Therapeutics Inc. for scientific presentation. CEA is a consultant to AstraZeneca and Mantra Bio; and received grant support from Sana. SB is an employee and stockholder of Travere Therapeutics Inc. PH is an employee and stockholder of Travere Therapeutics Inc. JI is an employee and stockholder of Travere Therapeutics Inc. RK is an employee and stockholder of Travere Therapeutics Inc. HJLH is a consultant to AbbVie, AstraZeneca, Bayer, Boehringer Ingelheim, Chinook, CSL Behring, Dimerix, Eli Lilly, Gilead, Janssen, Merck, Novo Nordisk, and Travere Therapeutics Inc.; research support for clinical trials from AstraZeneca, Boehringer Ingelheim, Janssen, and Novo Nordisk; and The George Institute for Global Health and George Clinical hold research contracts for trials in kidney disease. AM is a consultant to Travere Therapeutics Inc. through contract with JAMCO Pharma Consulting AB. ILN received honorarium for scientific presentation from AstraZeneca, Bayer, Novartis, and The George Institute for Global Health holds research contracts for trials in kidney disease. JR received consulting fees and grant support from Travere Therapeutics Inc. MNR is a clinical trial site PI for Chinook, Kaneka, Reata, River 3 Renal Corp., Sanofi, and Travere Therapeutics Inc.; is a consultant to Visterra; and is a data and safety monitoring board member for Advicenne. WR is an employee and stockholder of Travere Therapeutics Inc. HTra has served as a consultant to and/or a member of a data monitoring committee for Akebia, ChemoCentryx, Goldfinch Bio, Inc., Natera, Otsuka, Travere Therapeutics Inc., and Walden. HTri received honorarium for scientific work from AstraZeneca, Bayer, Calliditas, Chinook, Dimerix, GlaxoSmithKline, Novartis, Omeros, Roche, Travere Therapeutics Inc., and Visterra Otsuka; and The George Institute for Global Health holds research contracts for trials in kidney disease. VP received grants from Pfizer, which provided study drug and initial seed funding, during the conduct of the study; received grants from AbbVie for a clinical trial steering committee; received personal fees from Amgen for serving on an advisory board; serving on a clinical trial steering committee for Astellas; received personal fees from AstraZeneca, Boehringer, Ingelheim, Janssen, Novo Nordisk, and Novartis for serving on a steering committee, advisory committee, and scientific presentations; serves on a trial steering committee and advisory committee for Bayer; received personal fees from Chinook Therapeutics for serving on an advisory committee; serving on a data and safety monitoring committee for Dimerix; serving on the board of directors for George Clinical; serving on a steering committee and advisory committee for Gilead, GlaxoSmithKline, and Travere Therapeutics, Inc.; serving on an advisory committee for Medimmune; receiving personal fees from Mitsubishi Tanabe for scientific presentations; receiving personal fees from Mundipharma for advisory committee and scientific presentations; and serving on an advisory committee for Vifor Pharma; and holds research contracts with The George Institute for Global Health for trials in kidney disease.

## Supplementary Material

Supplementary File (PDF)

**Figure S1.** Patients receiving ACEi and ARB at screening at <50%, 50% to 80%, and >80% of MLD for patients on ACEi only, patients on ARB only, and patients on ACEi and ARB by (a) Asian geographic regions and (b) Non-Asian Geographic Regions.

**Table S1.** Baseline demographic characteristics and relevant medical history of patients who were randomized and received study drug in PROTECT by Asian and non-Asian geographic regions.

**Table S2.** Laboratory values at baseline in patients who were randomized and received study drug in PROTECT by Asian and non-Asian geographic regions.

**Table S3.** Medications at screening and baseline for patients who were randomized and received study drug in PROTECT by Asian and non-Asian geographic regions.

**Table S4.** The minimum daily doses for the most common ACEis and ARBs for study eligibility screening.

**Table S5.** Baseline demographic characteristics and relevant medical history of patients who were randomized and received study drug in PROTECT by Asian and non-Asian race within geographic regions.

**Table S6.** Laboratory values at baseline in patients who were randomized and received study drug in PROTECT by Asian and non-Asian race within geographic regions.

**Table S7.** Medications at screening and baseline for patients who were randomized and received study drug in PROTECT by Asian and non-Asian race within geographic regions.

**CONSORT Statement.**
