# Supplementary material for: IgA Nephropathy Patient Baseline Characteristics in the Sparsentan PROTECT Study
Source: Kidney Int Rep. 2023 Mar 4;8(5):1043–56. doi: 10.1016/j.ekir.2023.02.1086 (PMC10166729; doi:10.1016/j.ekir.2023.02.1086)
Supplement: Supplementary File (PDF) [file mmc1.pdf]

Supplemental Tables and Figure

Figure S1. Patients receiving ACEi and ARB at screening at <50%, 50% to 80%, and >80% of MLD for patients on ACEi only, patients on ARB only, and patients on ACEi and ARB by (a) Asian geographic regions and (b) Non-Asian Geographic Regions

a.

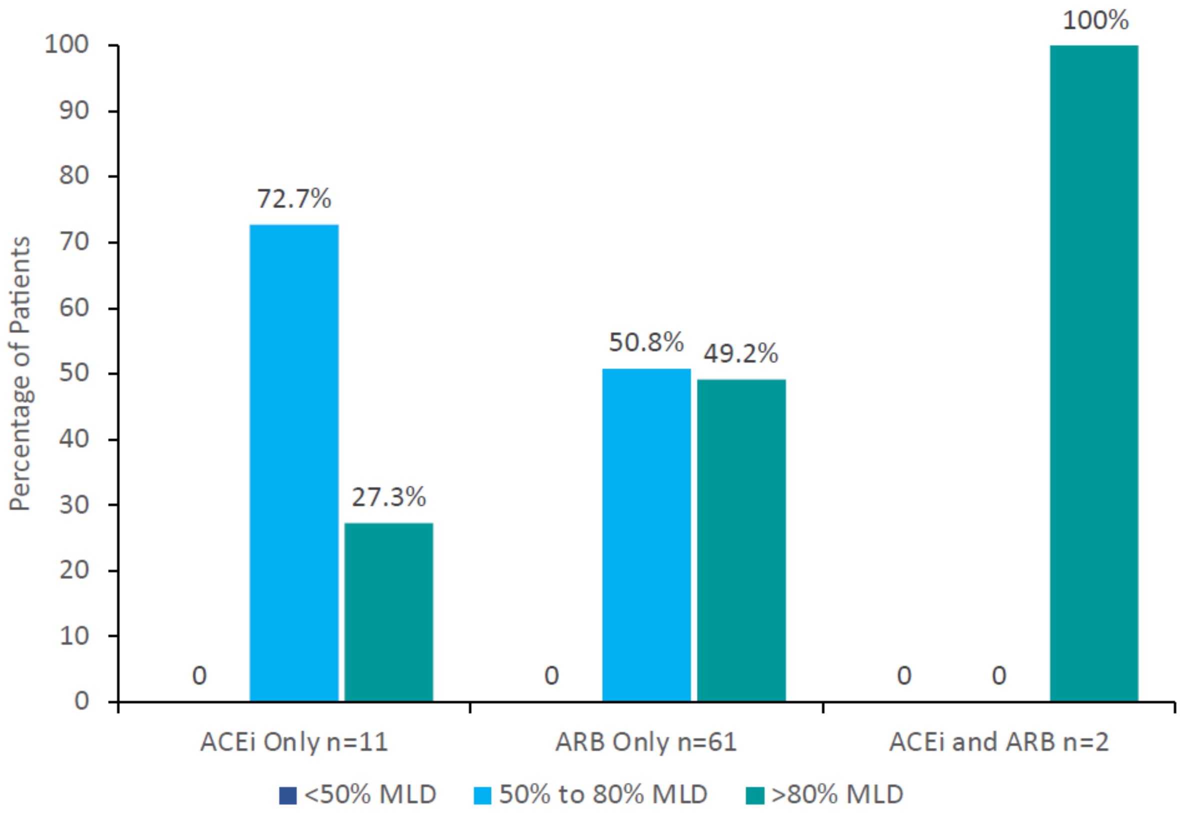

b.

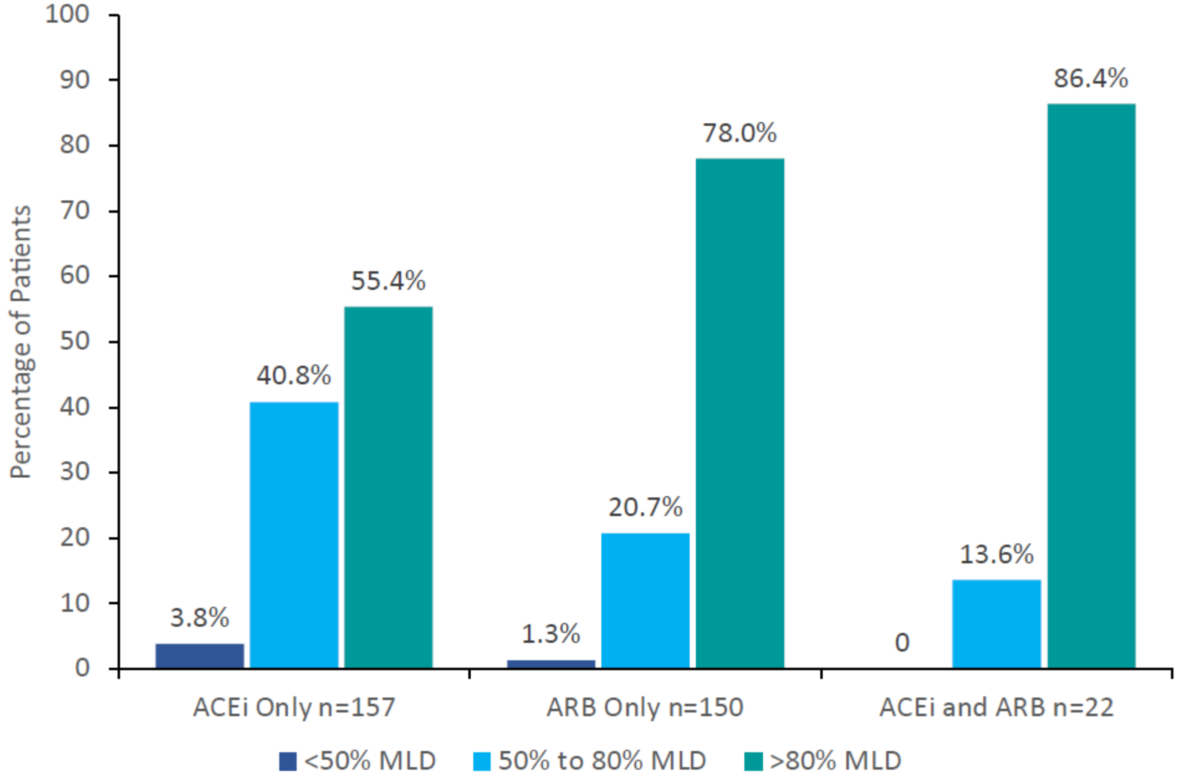

For patients with more than 1 record of MLD percentage and for patients taking both ACEi and ARB treatment, the highest percentage MLD was included.

Asian geographic regions include Hong Kong, Taiwan, and South Korea. Non-Asian geographic regions include all other countries.

ACEi, angiotensin converting enzyme inhibitor; ARB, angiotensin receptor blocker; MLD, maximum labeled dose.

Table S1. Baseline demographic characteristics and relevant medical history of patients who were randomized and received study drug in PROTECT by Asian and non-Asian geographic regions

Characteristic	Asian geographic regions (n=74)	Non-Asian geographic regions (n=330)
Age at informed consent, years	48.5 (40.0-56.0)	46.0 (36.0-56.0)
Sex		
Male	43 (58.1)	239 (72.4)
Female	31 (41.9)	91 (27.6)
Race[a]		
White	0	272 (82.4)
Asian	74 (100)	41 (12.4)
Black or African American	0	4 (1.2)
Other	0	13 (3.9)
Ethnicity		
Not Hispanic or Latino	74 (100)	294 (89.1)
Hispanic or Latino	0	33 (10.0)
Not reported	0	3 (0.9)
Age at IgAN diagnosis, years[b]	41.5 (33.0-51.0)	37.5 (29.0-49.0)
Time from initial kidney biopsy to informed consent, years[c]	3.0 (1.0-9.0)	4.0 (1.0-10.0)
History of diabetes and impaired fasting glucose	8 (10.8)	35 (10.6)
History of hypertension	43 (58.1)	266 (80.6)
Blood pressure, mmHg		
Systolic	124.1 ± 14.5	130.1 ± 13.0
Diastolic	80.1 ± 10.9	83.0 ± 10.5
BMI, kg/m^2	26.6 ± 4.6	28.9 ± 5.5

Data are given as n (%), median (IQR), or mean ± SD. Asian geographic regions include Hong Kong, Taiwan, and South Korea. Non-Asian geographic regions include all other countries.
BMI, body mass index; IgAN, immunoglobulin A nephropathy; IQR, interquartile range.
[a]Patients may have selected more than 1 race. “Other” race included American Indian or Alaskan Native, Native Hawaiian or Other Pacific Islander, and Other.
[b]Age at IgAN diagnosis is derived based on the year of IgAN diagnosis and year of birth.
[c]Time from initial biopsy is derived based on the year of the initial kidney biopsy and year of signed informed consent.

Table S2. Laboratory values at baseline in patients who were randomized and received study drug in PROTECT by Asian and non-Asian geographic regions

Characteristic	Asian geographic regions (n=74)	Non-Asian geographic regions (n=330)
UP/C, g/g	1.4 (0.9-1.9)	1.2 (0.8-1.8)
Urinary protein excretion, g/day	1.7 (1.2-2.5)	1.8 (1.3-2.8)
Nephrotic range proteinuria (>3.5 g/day)	8 (10.8)	41 (12.4)
UA/C, g/g	1.2 (0.8-1.6)	1.0 (0.7-1.5)
Urinary albumin excretion, mg/day	1465 (1087-2195)	1499 (1057-2288)
eGFR[a]		
Mean ± SD	59.3 ± 23.7	56.4 ± 24.0
Median (IQR)	51.5 (41.0-73.0)	50.0 (38.0-70.0)
eGFR		
≥90	12 (16.2)	39 (11.8)
≥60 to <90	16 (21.6)	81 (24.5)
≥45 to <60	19 (25.7)	75 (22.7)
≥30 to <45	25 (33.8)	117 (35.5)
≥15 to <30	2 (2.7)	18 (5.5)
Hemoglobin, g/L	133.6 ± 15.1	139.9 ± 15.9
Plasma lipid profile, mmol/L		
Total cholesterol	4.6 ± 1.0	5.0 ± 1.1
HDL cholesterol	1.3 ± 0.4	1.3 ± 0.4
LDL cholesterol	2.4 ± 0.8	2.9 ± 1.0
Triglycerides	1.9 ± 1.0	1.9 ± 1.1
Serum albumin, g/L		
Mean ± SD	40.6 ± 5.1	41.6 ± 3.5
Median (IQR)	41.5 (38.0-44.0)	42.0 (40.0-44.0)
Serum potassium, mmol/L	4.7 ± 0.4	4.6 ± 0.4
Serum creatinine, µmol/L	125.8 ± 42.1	138.4 ± 45.7
Serum cystatin C, mg/L	1.4 ± 0.4	1.5 ± 0.4
Hematuria/microscopic hematuria[b]	38 (51.4)	187 (56.7)
Urine creatinine, mg/dL	73.2 ± 30.9	74.8 ± 30.8
Urine sodium, mEq/L	78.6 ± 27.8	80.4 ± 32.7
ALT, U/L	22.5 ± 13.5	21.6 ± 10.2
AST, U/L	21.4 ± 8.8	21.1 ± 7.8

Data are given as n (%), median (IQR), or mean ± SD. Asian geographic regions include Hong Kong, Taiwan, and South Korea. Non-Asian geographic regions include all other countries.

ALT, alanine transaminase; AST, aspartate transferase; eGFR, estimated glomerular filtration rate in ml/min/1.73 m^2; HDL, high-density lipoprotein; IQR, interquartile range; LDL, low-density lipoprotein; SD, standard deviation; UA/C, urine albumin/creatinine ratio; UP/C, urine protein/creatinine ratio.

A central laboratory was used for all laboratory testing analyses.

[a]eGFR was determined using the Chronic Kidney Disease Epidemiology (CKD-EPI) formula.

[b]The assessment of macroscopic hematuria was not possible due to the use of a central laboratory, resulting in an unreliable analysis of macrohematuria due to the transport time and analysis delays.

Table S3. Medications at screening and baseline for patients who were randomized and received study drug in PROTECT by Asian and non-Asian geographic regions

Characteristic	Asian geographic regions (n=74)	Non-Asian geographic regions (n=330)
ACEi and ARB treatment at screening[a]		
ACEi only, n (%, % on MLD)	11 (14.9, 27.3)	157 (47.6, 54.1)
ARB only, n (%, % on MLD)	61 (82.4, 49.2)	150 (45.5, 78.0)
ACEi and ARB, n (%, % on MLD of both, % on MLD of either)	2 (2.7, 100, 100)	22 (6.7, 31.8, 86.4)
MLD of ACEi or ARB, n (%)	35 (47.3)	221 (67.0)
Baseline medication use, n (%)[b]		
Antihypertensive medications[c]	27 (36.5)	147 (44.6)
Diuretics	5 (6.8)	57 (17.3)
Beta-blockers	9 (12.2)	46 (13.9)
Alpha-blockers	5 (6.8)	17 (5.2)
Calcium channel blockers	20 (27.0)	89 (27.0)
Other	1 (1.4)	22 (6.7)
≥2 antihypertensive medications at baseline (excluding RAASi medications)	8 (10.8)	53 (16.1)
Number of antihypertensive medications per patient (including RAASi medications)		
Mean ± SD	1.4 ± 0.7	1.6 ± 0.9
Median (IQR)	1.0 (1-2)	1.0 (1-2)
Lipid-lowering medications	50 (67.6)	173 (52.4)

Asian geographic regions include Hong Kong, Taiwan, and South Korea. Non-Asian geographic regions include all other countries.

ACEi, angiotensin converting enzyme inhibitor; ARB, angiotensin receptor blocker; IQR, interquartile range; MLD, maximum labeled dose; RAASi, renin-angiotensin-aldosterone system inhibitors; SD, standard deviation.

[a]ACEi and ARB treatment at screening; RAASi were prohibited during the study. Each "% on MLD" is based on the related n-value of patients receiving ACEi only, ARB only, or ACEi and ARB.

[b]Baseline medications were started prior to randomization (Day 1) and continued after the initial dose of study medication.

[c]Antihypertensive medications exclude ACEis, ARBs, aldosterone blockers, and aliskiren.

Table S4. Baseline demographic characteristics and relevant medical history of patients who were randomized and received study drug in PROTECT by Asian and non-Asian race within geographic regions

Characteristic	Asian geographic regions	Non-Asian geographic regions	
	Asian race (n=74)	Asian race (n=42)	Non-Asian race (n=288)
Age at informed consent, years	48.5 (40.0-56.0)	45.5 (38.0-57.0)	46.0 (36.0-56.0)
Sex			
Male	43 (58.1)	20 (47.6)	219 (76.0)
Female	31 (41.9)	22 (52.4)	69 (24.0)
Race[a]			
White	0	0	272 (94.4)
Asian	74 (100)	41 (97.6)	0
Black or African American	0	0	4 (1.4)
Other	0	1 (2.4)	12 (4.2)
Ethnicity			
Not Hispanic or Latino	74 (100)	42 (100)	252 (87.5)
Hispanic or Latino	0	0	33 (11.5)
Not reported	0	0	3 (1.0)
Age at IgAN diagnosis, years[b]	41.5 (33.0-51.0)	37.0 (31.0-46.0)	38.0 (29.0-49.0)
Time from initial kidney biopsy to informed consent, years[c]	3.0 (1.0-9.0)	4.5 (2.0-9.0)	4.0 (1.0-10.0)
History of diabetes or impaired fasting glucose	8 (10.8)	6 (14.3)	29 (10.1)
History of hypertension	43 (58.1)	26 (61.9)	240 (83.3)
Blood pressure, mmHg			
Systolic	124.1 ± 14.6	124.5 ± 9.9	130.9 ± 13.2
Diastolic	80.1 ± 10.9	81.1 ± 9.0	83.2 ± 10.7
BMI, kg/m^2	26.6 ± 4.6	27.9 ± 5.0	29.0 ± 5.6

Data are given as n (%), median (IQR), or mean ± SD. Asian geographic regions include Hong Kong, Taiwan, and South Korea. Non-Asian geographic regions include all other countries. For patients who selected multiple races, if one of the races was Asian, the patient was included as Asian.
BMI, body mass index; IgAN, immunoglobulin A nephropathy; IQR, interquartile range.
[a]Patients may have selected more than 1 race. "Other" race included American Indian or Alaskan Native, Native Hawaiian or Other Pacific Islander, and Other.
[b]Age at IgAN diagnosis is derived based on the year of IgAN diagnosis and year of birth.
[c]Time from initial biopsy is derived based on the year of the initial kidney biopsy and year of signed informed consent.

Table S5. Laboratory values at baseline in patients who were randomized and received study drug in PROTECT by Asian and non-Asian race within geographic regions

**Characteristic**	**Asian geographic regions**	**Non-Asian geographic regions**	
	**Asian race (n=74)**	**Asian race (n=42)**	**Non-Asian race (n=288)**
UP/C, g/g	1.4 (0.9-1.9)	1.3 (0.9-2.1)	1.2 (0.8-1.8)
Urinary protein excretion, g/day	1.7 (1.2-2.5)	1.7 (1.2-2.4)	1.8 (1.3-2.9)
Nephrotic range proteinuria (>3.5 g/day)	8 (10.8)	4 (9.5)	37 (12.8)
UA/C, g/g	1.2 (0.8-1.6)	1.0 (0.7-1.7)	1.0 (0.7-1.4)
Urinary albumin excretion, mg/day	1465 (1087-2195)	1485 (1034-1834)	1508 (1059-2376)
eGFR[a]			
Mean ± SD	59.3 ± 23.7	57.5 ± 23.9	56.3 ± 24.1
Median (IQR)	51.5 (41.0-73.0)	50.5 (38.0-72.0)	50.0 (38.0-70.0)
eGFR			
≥90	12 (16.2)	6 (14.3)	33 (11.5)
≥60 to <90	16 (21.6)	11 (26.2)	70 (24.3)
≥45 to <60	19 (25.7)	8 (19.0)	67 (23.3)
≥30 to <45	25 (33.8)	15 (35.7)	102 (35.4)
≥15 to <30	2 (2.7)	2 (4.8)	16 (5.6)
Hemoglobin, g/L	133.6 ± 15.1	134.3 ± 14.4	140.7 ± 16.0
Plasma lipid profile, mmol/L			
Total cholesterol	4.6 ± 1.0	5.1 ± 1.2	5.0 ± 1.1
HDL cholesterol	1.3 ± 0.4	1.3 ± 0.4	1.3 ± 0.4
LDL cholesterol	2.4 ± 0.8	3.0 ± 1.0	2.9 ± 1.1
Triglycerides	1.9 ± 1.0	2.1 ± 1.1	1.9 ± 1.1
Serum albumin, g/L			
Mean ± SD	40.6 ± 5.1	41.0 ± 4.3	41.7 ± 3.4
Median (IQR)	41.5 (38.0-44.0)	42.0 (40.0-44.0)	42.0 (40.0-44.0)
Serum potassium, mmol/L	4.7 ± 0.4	4.4 ± 0.5	4.7 ± 0.4
Serum creatinine, μmol/L	125.8 ± 42.1	128.0 ± 44.8	139.9 ± 45.7
Serum cystatin C, mg/L	1.4 ± 0.4	1.5 ± 0.4	1.5 ± 0.4
Hematuria/microscopic hematuria[b]	38 (51.4)	19 (45.2)	168 (58.3)
Urine sodium, mEq/L	78.6 ± 27.8	69.1 ± 25.3	82.0 ± 33.3

eGFR, estimated glomerular filtration rate in ml/min/1.73m^2; HDL, high-density lipoprotein; IQR, interquartile range; LDL, low-density lipoprotein; SD, standard deviation; UA/C, urine albumin/creatinine ratio; UP/C, urine protein/creatinine ratio.

Data are given as n (%), median (IQR), or mean ± SD. A central laboratory was used for all laboratory testing analyses.

[a]eGFR was determined using the Chronic Kidney Disease Epidemiology (CKD-EPI) formula.

[b]The assessment of macroscopic hematuria was not possible due to the use of a central laboratory, resulting in an unreliable analysis of macrohematuria due to the transport time and analysis delays.

Table S6. Medications at screening and baseline for patients who were randomized and received study drug in PROTECT by Asian and non-Asian race within geographic regions

**Characteristic**	**Asian geographic regions**	**Non-Asian geographic regions**	
	**Asian race (n=74)**	**Asian race (n=42)**	**Non-Asian race (n=288)**
ACEi and ARB treatment at screening, n (%)[a]			
ACEi only, n (%, % on MLD)	11 (14.9, 27.3)	17 (40.5, 41.2)	140 (48.6, 55.7)
ARB only, n (%, % on MLD)	61 (82.4, 49.2)	23 (54.8, 87.0)	127 (44.1, 76.4)
ACEi and ARB, n (%, % on MLD of both, % on MLD of either)	2 (2.7, 100, 100)	2 (4.8, 0, 100)	20 (6.9, 35.0, 85.0)
MLD of ACEi or ARB, n (%)	35 (47.3)	29 (69.0)	192 (66.7)
Baseline medication use, n (%)[b]			
Antihypertensive medications[c]	27 (36.5)	14 (33.3)	133 (46.2)
Diuretics	5 (6.8)	5 (11.9)	52 (18.1)
Beta-blockers	9 (12.2)	5 (11.9)	41 (14.2)
Alpha-blockers	5 (6.8)	0	17 (5.9)
Calcium channel blockers	20 (27.0)	10 (23.8)	79 (27.4)
Other	1 (1.4)	1 (2.4)	21 (7.3)
≥2 antihypertensive medications at baseline (excluding RAASi medications)	8 (10.8)	5 (11.9)	48 (16.7)
Number of antihypertensive medications per patient (including RAASi medications)			
Mean ± SD	1.4 ± 0.7	1.6 ± 0.9	1.6 ± 0.9
Median (IQR)	1.0 (1-2)	1.0 (1-2)	1.0 (1-2)
Lipid-lowering medications	50 (67.6)	24 (57.1)	149 (51.7)

Data are given as n (%), median (IQR), or mean ± SD. Asian geographic regions include Hong Kong, Taiwan, and South Korea. Non-Asian geographic regions include all other countries. For patients who selected multiple races, if one of the races was Asian, the patient was included as Asian.

ACEi, angiotensin converting enzyme inhibitor; ARB, angiotensin receptor blocker; IQR, interquartile range; MLD, maximum labeled dose; RAASi, renin-angiotensin-aldosterone system inhibitors; SD, standard deviation.

[a]ACEi and ARB treatment at screening; RAASi were prohibited during the study. Each "% on MLD" is based on the related n-value of patients receiving ACEi only, ARB only, or ACEi and ARB.

[b]Baseline medications were started prior to randomization (Day 1) and continued after the initial dose of study medication.

[c]Antihypertensive medications exclude ACEis, ARBs, aldosterone blockers, and aliskiren.

Table S7. The minimum daily doses for the most common ACEis and ARBs for study eligibility screening

ACEi	Minimum daily dose at screening	ARB	Minimum daily dose at screening
Benazepril	20 mg	Candesartan	16 mg
Captopril	75 mg	Eprosartan	300 mg
Enalapril	20 mg	Irbesartan	150 mg
Fosinopril	20 mg	Losartan	50 mg
Moexipril	15 mg	Valsartan	160 mg
Perindopril	4 mg	Telmisartan	40 mg
Quinapril	20 mg	Olmesartan	20 mg
Ramipril	5 mg	Azilsartan	40 mg
Trandolapril	2 mg		
Lisinopril	20 mg		
Zofenopril	30 mg		
Cilazapril	5 mg		
Delapril	60 mg		
Imidapril	10 mg		

ACEi, angiotensin converting enzyme inhibitor; ARB, angiotensin receptor blocker; MLD, maximum labeled dose.

Values in the table represent 50% of the MLD of the drugs in most participating countries. The values are considered approximately equivalent to the minimum daily dose of the active comparator for the treatment phase of PROTECT (ie, irbesartan 150 mg/day). If a patient is on a combination of an ACEi and an ARB, the sum of the individual doses (as a percentage of the MLD on the table) should be at least 50% (eg, 2.5 mg/day ramipril [25%] + 25 mg losartan [25%] = 50% in total).

CONSORT 2010 checklist of information to include when reporting a randomised trial*

Section/Topic	Item No	Checklist item	Reported on page No
**Title and abstract**			
	1a	Identification as a randomised trial in the title	Not in title as this is an aggregated baseline characteristics paper and word limit prevented full trial description in title
	1b	Structured summary of trial design, methods, results, and conclusions (for specific guidance see CONSORT for abstracts)	3
**Introduction**			
Background and objectives	2a	Scientific background and explanation of rationale	4-5
	2b	Specific objectives or hypotheses	5
**Methods**			
Trial design	3a	Description of trial design (such as parallel, factorial) including allocation ratio	5
	3b	Important changes to methods after trial commencement (such as eligibility criteria), with reasons	NA
Participants	4a	Eligibility criteria for participants	5-6
	4b	Settings and locations where the data were collected	5/8
Interventions	5	The interventions for each group with sufficient details to allow replication, including how and when they were actually administered	NA (baseline focus)
Outcomes	6a	Completely defined pre-specified primary and secondary outcome measures, including how and when they were assessed	7
	6b	Any changes to trial outcomes after the trial commenced, with reasons	NA (baseline focus)
Sample size	7a	How sample size was determined	5

	7b	When applicable, explanation of any interim analyses and stopping guidelines	7
Randomisation:			
Sequence generation	8a	Method used to generate the random allocation sequence	5
	8b	Type of randomisation; details of any restriction (such as blocking and block size)	5
Allocation concealment mechanism	9	Mechanism used to implement the random allocation sequence (such as sequentially numbered containers), describing any steps taken to conceal the sequence until interventions were assigned	5
Implementation	10	Who generated the random allocation sequence, who enrolled participants, and who assigned participants to interventions	5
Blinding	11a	If done, who was blinded after assignment to interventions (for example, participants, care providers, those assessing outcomes) and how	5
	11b	If relevant, description of the similarity of interventions	5
Statistical methods	12a	Statistical methods used to compare groups for primary and secondary outcomes	NA (baseline focus)
	12b	Methods for additional analyses, such as subgroup analyses and adjusted analyses	NA (baseline focus)
**Results**			
Participant flow (a diagram is strongly recommended)	13a	For each group, the numbers of participants who were randomly assigned, received intended treatment, and were analysed for the primary outcome	8
	13b	For each group, losses and exclusions after randomisation, together with reasons	8
Recruitment	14a	Dates defining the periods of recruitment and follow-up	NA (trial in progress)
	14b	Why the trial ended or was stopped	NA (trial in progress)
Baseline data	15	A table showing baseline demographic and clinical characteristics for each group	23-28
Numbers analysed	16	For each group, number of participants (denominator) included in each analysis and whether the analysis was by original assigned groups	NA (baseline focus)
Outcomes and estimation	17a	For each primary and secondary outcome, results for each group, and the estimated effect size and its precision (such as 95% confidence interval)	NA (baseline focus)
	17b	For binary outcomes, presentation of both absolute and relative effect sizes is recommended	NA (baseline focus)
Ancillary analyses	18	Results of any other analyses performed, including subgroup analyses and adjusted analyses, distinguishing pre-specified from exploratory	10 and Supplemental

			tables
Harms	19	All important harms or unintended effects in each group (for specific guidance see CONSORT for harms)	NA (baseline focus)
**Discussion**			
Limitations	20	Trial limitations, addressing sources of potential bias, imprecision, and, if relevant, multiplicity of analyses	15 (baseline focus)
Generalisability	21	Generalisability (external validity, applicability) of the trial findings	14-16 (baseline focus)
Interpretation	22	Interpretation consistent with results, balancing benefits and harms, and considering other relevant evidence	12-16
**Other information**			
Registration	23	Registration number and name of trial registry	16
Protocol	24	Where the full trial protocol can be accessed, if available	NA (trial ongoing)
Funding	25	Sources of funding and other support (such as supply of drugs), role of funders	16

*We strongly recommend reading this statement in conjunction with the CONSORT 2010 Explanation and Elaboration for important clarifications on all the items. If relevant, we also recommend reading CONSORT extensions for cluster randomised trials, non-inferiority and equivalence trials, non-pharmacological treatments, herbal interventions, and pragmatic trials. Additional extensions are forthcoming: for those and for up to date references relevant to this checklist, see www.consort-statement.org.
